# Comprehensive analysis of mutations and clonal evolution patterns in a cohort of patients with cytogenetically normal acute myeloid leukemia

**DOI:** 10.1186/s13039-021-00561-2

**Published:** 2021-09-24

**Authors:** Yuslina Mat Yusoff, Fadly Ahid, Zahidah Abu Seman, Julia Abdullah, Nor Rizan Kamaluddin, Ezalia Esa, Zubaidah Zakaria

**Affiliations:** 1 Hematology Unit, Cancer Research Centre, Institute for Medical Research, National Institutes of Health, Ministry of Health Malaysia, 40170 Shah Alam, Selangor Malaysia; 2 Centre for Medical Laboratory Technology Studies, Faculty of Health Sciences, Universiti Teknologi MARA, 42300 Puncak Alam, Selangor Malaysia

**Keywords:** Acute myeloid leukemia, Clonal evolution, Mutation, Relapse, Whole exome sequencing

## Abstract

**Background:**

Relapsed acute myeloid leukemia (AML) is associated with the acquisition of additional somatic mutations which are thought to drive phenotypic adaptability, clonal selection and evolution of leukemic clones during treatment. We performed high throughput exome sequencing of matched presentation and relapsed samples from 6 cytogenetically normal AML (CN-AML) patients treated with standard remission induction chemotherapy in order to contribute with the investigation of the mutational landscape of CN-AML and clonal evolution during AML treatment.

**Result:**

A total of 24 and 32 somatic variants were identified in presentation and relapse samples respectively with an average of 4.0 variants per patient at presentation and 5.3 variants per patient at relapse, with SNVs being more frequent than indels at both disease stages. All patients have somatic variants in at least one gene that is frequently mutated in AML at both disease presentation and relapse, with most of these variants are classic AML and recurrent hotspot mutations including *NPM1* p.W288fs, *FLT3*-ITD, *NRAS* p.G12D and *IDH2* p.R140Q. In addition, we found two distinct clonal evolution patterns of relapse: (1) a leukemic clone at disease presentation acquires additional mutations and evolves into the relapse clone after the chemotherapy; (2) a leukemic clone at disease presentation persists at relapse without the addition of novel somatic mutations.

**Conclusions:**

The findings of this study suggest that the relapse-initiating clones may pre-exist prior to therapy, which harbor or acquire mutations that confer selective advantage during chemotherapy, resulting in clonal expansion and eventually leading to relapse.

## Background

Cytogenetic analysis has been used for more than three decades to define the molecular pathogenesis of acute myeloid leukemia (AML), and remains as the first-tier screening for AML classification. Recurrent chromosomal structural variations such as t(8;21), inv(16), t(15;17), del(5) and del(7) are established diagnostic and prognostic markers suggesting that acquired genomic abnormalities play an essential role in leukemogenesis [1]. However, nearly half of adult AML cases (40–47%) and about 15–30% of pediatric AML cases have a normal karyotype lacking recurrent structural abnormalities [2, 3]. Somatic point mutations affecting numerous genes have been described in AML at presentation, many of which are pathogenic and prognostic, including *RUNX1* [4], *NPM1* [5], *NRAS/KRAS* [6] and *CEBPA* [7]. High throughput sequencing technology has intensified the search for somatic mutations in AML, leading to the discovery of novel mutations in established pathways, such as *RAS* [8], and identified new pathways through whole genome-based approaches [9]. This approach has also been applied to the study of relapsed leukemia, successfully identifying relapse-specific coding sequence mutations affecting *ETV6* and *MYO18B* [10].

Cancer is an evolutionary process, as evidenced by the accumulation of somatic mutations in cancer cells as a result of continuous mutation acquisition during disease progression. Most mutations at disease presentation in AML are thought to be acquired after initiating genetic lesions such as t(8;21) and t(15;17), although a small number of pre-existing mutations are already present before cells acquire these advantageous initiating mutations. There are some evidences that initiating events promote mutagenesis, predisposing cells to the acquisition of additional somatic mutations [11–14] which may contribute to genomic instability in a cancer genome. Genomic instability can increase the mutation rate in the cancer genome through many different mechanisms, which play an important role in cancer evolution.

While chemotherapy with cytotoxic agents may eliminate cancer cells with dominant clones during remission induction treatment, the majority of AML patients experience disease relapse, which is difficult to treat. The mechanisms driving relapse evolution remain unclear although there is evidence that relapse is driven by novel mutations acquired after the chemotherapy. High throughput sequencing of matched presentation and relapse AML samples have revealed two major patterns of AML relapse evolution [10, 15–17]. Firstly, a leukemic clone at disease presentation acquires additional mutations and evolves into the relapse clone after the chemotherapy and secondly, the pre-leukemic clone from the founding clone acquires additional mutations and evolves into a relapse clone. Importantly, both patterns of relapse are defined by the acquisition of additional somatic mutations.

Therefore, it is thought that there are two major underlying mechanisms driving mutagenesis in relapsing AML. Firstly, the genomic instability of the leukemic clone is a major cause of mutagenesis and contributes to genetic heterogeneity in AML. Secondly, chemotherapy agents used in induction chemotherapy contribute to relapsed AML since most chemotherapy agents are genotoxic, which may lead to mutation. Collectively, both mechanisms are predicted to be responsible for the etiology of relapse-driver mutations. In this study, whole exome sequencing of matched presentation and relapse AML samples was performed to map changes in the mutational landscape between presentation and relapse AML in order to determine the clonal origins of relapsed AML and clonal evolution during treatment of AML in order to better understand disease evolution and heterogeneity in AML.

## Results

### Identification of somatic variants

Somatic variants including nonsynonymous single nucleotide variant (SNV, both missense and nonsense) and small insertion/deletion (indel, both in-frame and frameshift), as well as splice site variants were identified following several layers of filters using Ingenuity Variant Analysis software and after exclusion of variants with benign and uncertain significance (Table 1). Two mutations at presentation, *NPM1* p.W288fs (Patient 1), *FLT3*-ITD (Patient 1) and one mutation at relapse, *NPM1* p.W288fs (Patient 5) detected from initial RT-PCR screening were not detected by exome sequencing. In total, 24 and 32 variants were identified in presentation and relapse samples respectively (Table 2), with an average of 4.0 variants per patient at presentation (range 3–6) and 5.3 variants per patient at relapse (range 3–8). In terms of type of variant, SNVs were more common than indels at both presentation and relapse with 9 indels and 15 SNVs identified at presentation and 12 indels and 20 SNVs identified at relapse.

**Table 1:** Somatic variants identified from whole exome sequencing

Patient	Gene	Reference allele	Variant allele	Variation type	Gene region	AA change	Presentation		Relapse		Frequency in COSMIC (%)
							Read depth	VAF (%)	Read depth	VAF (%)	
1	*PTPRQ*	GGGT		Deletion	Exonic	NM_001145026.1; c.6452_6453 + 2delGGGT;	–	–	103	17.5	0.0
	*RB1*	AC		Frameshift deletion	Exonic	p.K123fs*39	104	15.4	–	–	0.0
	*RB1*		ATAC	Frameshift insertion	Exonic	p.P121fs*4	103	16.5	–	–	0.0
	*RB1*	T		Frameshift deletion	Exonic	p.Y120fs*8	99	17.2	–	–	0.0
	*RB1*	TTG		In-frame deletion	Exonic	p.A117_I118delinsV	99	17.2	–	–	0.0
	*RB1*	A		Frameshift deletion	Exonic	p.L116fs*12	95	17.9	–	–	0.0
	*RB1*	AATC		Frameshift deletion	Exonic	p.R114fs*13	98	17.4	–	–	0.0
	*SELE*	C	T	SNV	Splice site	NM_000450.2; c.902-1G>A	113	55.8	111	55.9	0.0
2	*ADAM9*		TTTT	Frameshift insertion	Exonic	NM_003816.2; c.1883_1884insTTTT; p.C629fs*3	43	18.6	–	–	0.0
	*APOB*	G	A	Nonsynonymous SNV	Exonic	NM_000384.2; c.10,579 C>T; p.R3527W	278	45.0	247	40.9	0.0
	*DNAH5*	T		Deletion	Splice site	NM_001369.2; c.2578-2delA	–	–	17	17.7	0.0
	*NRAS*	C	T	Nonsynonymous SNV	Exonic	NM_002524.4; c.35G>A; p.G12D	390	14.4	434	33.4	29.1
3	*CEBPA*		CTG	In-frame insertion	Exonic	NM_004364.4; c.934_936dupCAG; p.Q312dup	283	87.6	268	47.0	2.1
	*CFTR*	G	A	Nonsynonymous SNV	Exonic	NM_000492.3; c.1865G>A; p.G622D	103	50.5	94	56.4	0.0
	*MUTYH*	C	A	Stop gain SNV	Exonic	NM_012222.2 c.1435G>T; p.E479*	152	47.4	126	48.4	0.0
4	*ESRRB*	C	T	Nonsynonymous SNV	Exonic	NM_004452.3; c.1144 C>T; p.R382C	202	49.5	236	48.7	0.0
	*FLT3*	G	A	Nonsynonymous SNV	Exonic	NM_004119.2; c.1577 C>T; p.T526M	419	48.5	385	48.8	0.0
	*HBB*	AAAG		Frameshift deletion	Exonic	NM_000518.4; c.126_129delCTTT; p.F42fs*19	241	50.2	263	39.5	0.0
	*LFNG*		GATG	Frameshift insertion	Exonic	NM_001166355.1; c.163_166dupGATG; p.E56fs*2	104	50.0	116	35.3	0.0
	*MAP3K20*	A	T	SNV	Splice site	c.416-2 A>T	–	–	66	15.2	0.0
	*MAP3K20*	G	T	SNV	Splice site	c.416-1G>T	–	–	66	15.2	0.0
5	*ESRRB*	C	T	Nonsynonymous SNV	Exonic	NM_004452.3; c.1144 C>T; p.R382C	218	48.6	216	50.5	0.0
	*EYS*	C	A	Stop gain SNV	Exonic	c.8170G>T; p.E2724*	155	57.4	153	54.3	0.0
	*GUCY2D*	C	T	Nonsynonymous SNV	Exonic	NM_000180.3; c.164 C>T; p.T55M	75	66.7	64	53.1	0.0
	*IDH2*	C	T	Nonsynonymous SNV	Exonic	NM_001290114.1; c.419G>A; p.R140Q	124	38.7	115	26.1	42.9
	*NPM1*		TCTG	Frameshift insertion	Exonic	NM_002520.6; c.860_863dupTCTG; p.W288fs	–	–	17	41.2	36.3
	*SLC12A1*	C	A	Stop gain SNV	Exonic	c.1584 C>A; p.Y528*	204	55.4	146	38.4	0.0
	*WRN*	AGGG		Deletion	Splice site	NM_000553.5; c.840-2_841delAGGG;	–	–	31	19.4	0.0
	*WRN*		TTTT	Frameshift insertion	Exonic	NM_000553.5; c.844_845insTTTT; p.S282fs*24	–	–	31	19.4	0.0
6	*ATM*	G	T	Nonsynonymous SNV	Splice site	NM_000051.3; c.497-1G>T	–	–	18	33.3	0.0
	*ATRX*	G	A	Stop gain SNV	Exonic	c.7219 C>T p.R2407*	–	–	120	44.2	14.29
	*CEBPA*		TTGGCCTT	Frameshift insertion	Exonic	NM_004364.4; c.929_930insAAGGCCAA p.Q346fs*10	303	42.9	349	49.6	0.0
	*CEBPA*	CTCCA		Frameshift deletion	Exonic	NM_004364.4; c.923_927delTGGAG; p.V343fs*11	329	43.5	350	47.4	0.0
	*MPST*	C	A	Stop gain SNV	Exonic	c.52G>T; p.E18*	184	51.1	193	49.7	0.0
	*RYR2*	C	T	Nonsynonymous SNV	Exonic	NM_001035.2; c.13,739 C>T; p.T4580M	–	–	29	51.7	0.0
	*TBXAS1*	C	T	Stop gain SNV	Exonic	c.997 C>T; p.R333*	148	41.2	150	52.0	0.0
	*VWF*	G	A	Nonsynonymous SNV	Exonic	NM_000552.4; c.4195 C>T; p.R1399C	161	40.4	154	37.7	0.0

**Table 2:** Number of somatic variants at presentation and relapse

Patient	Presentation			Relapse		
	Indel	SNV	Total no. of variants	Indel	SNV	Total no. of variants
1	2	1	3	3	1	4
2	1	2	3	1	2	3
3	1	2	3	1	2	3
4	2	2	4	2	4	6
5	1	5	6	3	5	8
6	2	3	5	2	6	8
Total no. of variants	9	15	24	12	20	32

**Fig. 1:**
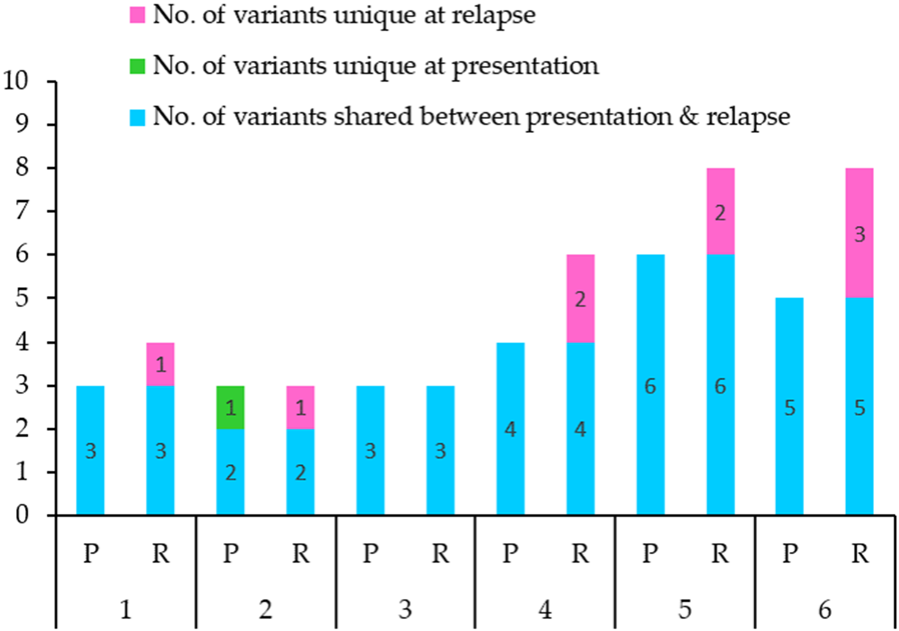
Total number of somatic variants specific to presentation or relapse and somatic variants shared at both disease stages

A total of 23 shared variants occurred at both presentation and relapse (Fig. 1). Moreover, 1 variant were presentation-specific, and 9 variants were relapse-specific. Additional somatic variants were acquired in five relapse samples (Patient 1, 2, 4, 5 and 6) which gained between 1 and 3 new variants when compared to their corresponding presentation samples. Two patients had the same number of variants at both presentation and relapse samples (Patient 2 and 3), of which 1 patient (Patient 2) acquired and lost equivalent numbers of variants.

### Evaluation of somatic variants at presentation and relapse

**Fig. 2:**
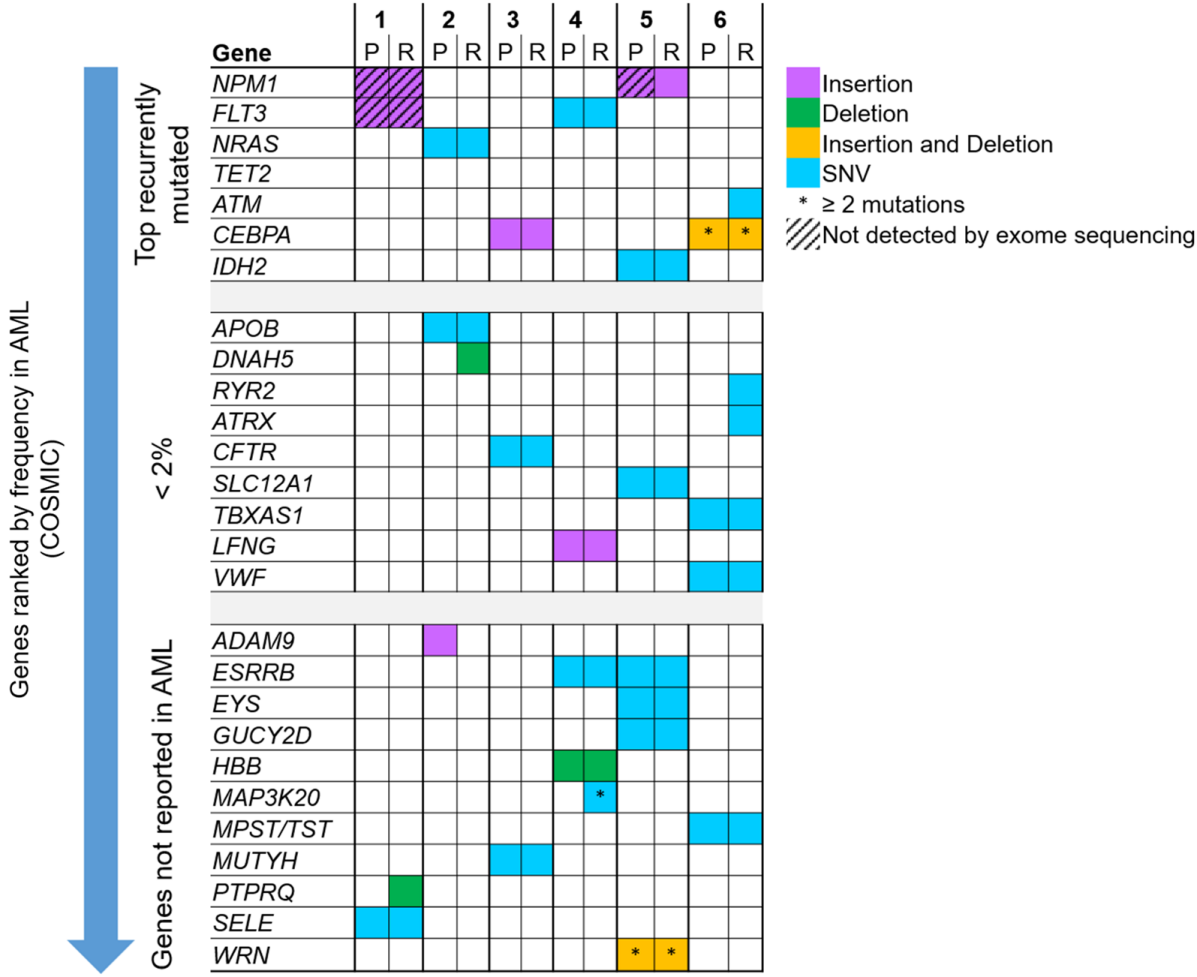
Mutational spectrum of somatic variants at presentation and relapse characterized according to frequency in AML as reported in COSMIC. The mutated genes identified in each patient were ranked according to frequency of genes implicated in AML as reported in the COSMIC database

The filtered somatic variants were further evaluated to define the oncogenic effect of the variants in determining their potential importance as AML driver mutations via interrogation of the COSMIC database. The mutated genes were ranked according to frequency of genes implicated in AML as reported in the COSMIC database regardless of type and position of the variants in genes, as either classical types of variants, or previously reported and/or recurrent hotspots variants, or novel variants (Fig. 2). Somatic variants in recurrently mutated genes in AML (*NPM1*, *FLT3*, *NRAS*, *ATM*, *CEBPA*, *IDH2*) were identified in all patients. Six of these variants are classic AML driver and recurrent hotspot mutations whereas variants such as *FLT3* p.T526M (Patient 4), *ATM* c.497-1G>T (Patient 6), *CEBPA* p.Q346fs*10 (Patient 6) and *CEBPA* p.V343fs*11 (Patient 6) were not previously reported in AML (Table 1). In addition to somatic variants in recurrently mutated genes, there were 9 mutated genes that have been previously reported in a small number of AML cases in COSMIC (less than 2%) and an additional 11 mutations in genes not previously reported in AML.

**Fig. 3:**
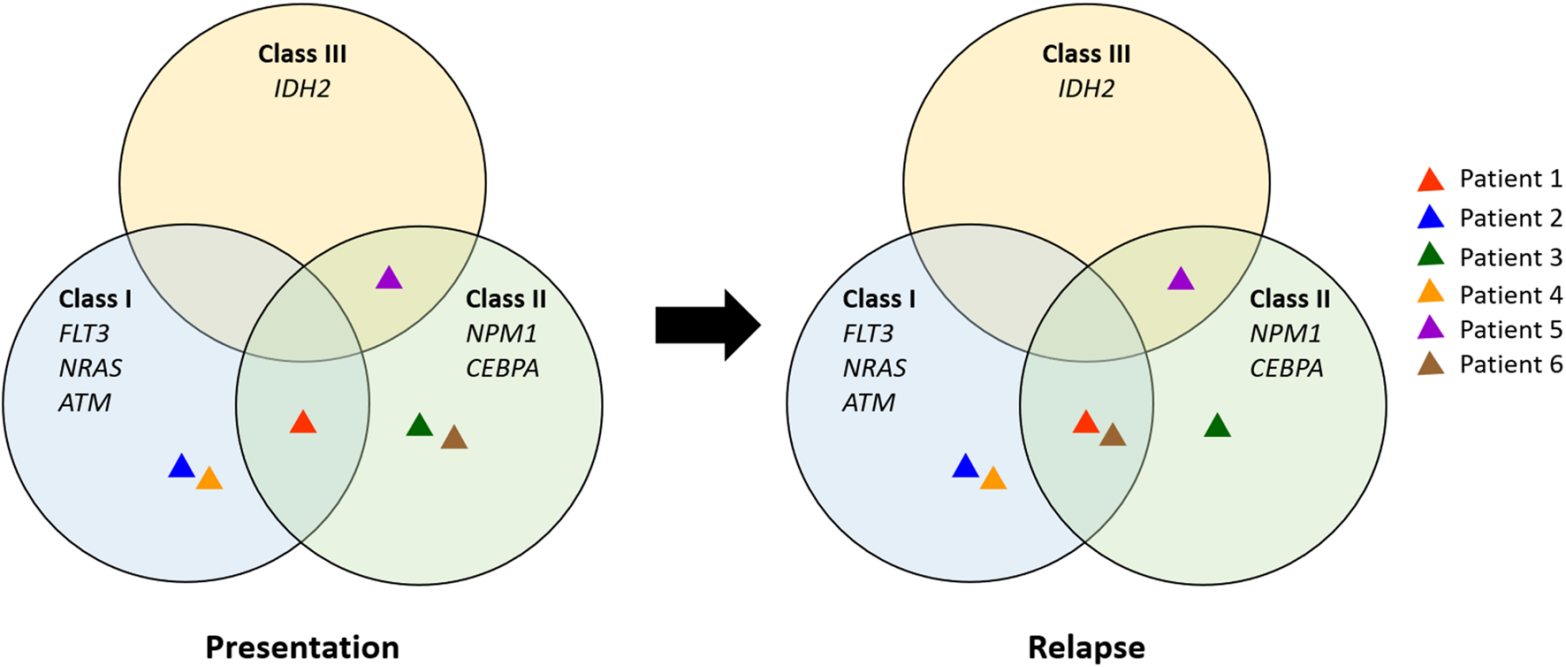
Classification of somatic variants at presentation and relapse samples based on their known or predicted role in leukemogenesis

To further analyze identified mutations, the mutated genes were clustered into three groups based on their putative effect that may relevant for leukemic transformation according to existing model of leukemogenesis [18, 19]: (1) mutations affecting genes that contribute to cell proliferation (class I mutations); (2) mutations affecting genes involved in myeloid differentiation (class II mutations); and (3) mutations affecting genes involved in epigenetic modification (class III mutations) (Fig. 3). All patients have mutations in at least one gene that either affect cell proliferation, are involved in myeloid differentiation or involved in epigenetic modification at presentation, with 1 patient (Patient 1) having both class I and II mutations at presentation. At relapse, acquisition of both classes of mutations were observed in 2 patients (Patient 1 and 6), where Patient 6 that lack complementary class of mutation at presentation acquired a class I mutation at relapse. In contrast, the remaining 20 mutated genes identified in this cohort have yet unknown functions in leukemogenesis.

### Correlation of presentation variant allele fraction (VAF) and relapse

**Fig. 4:**
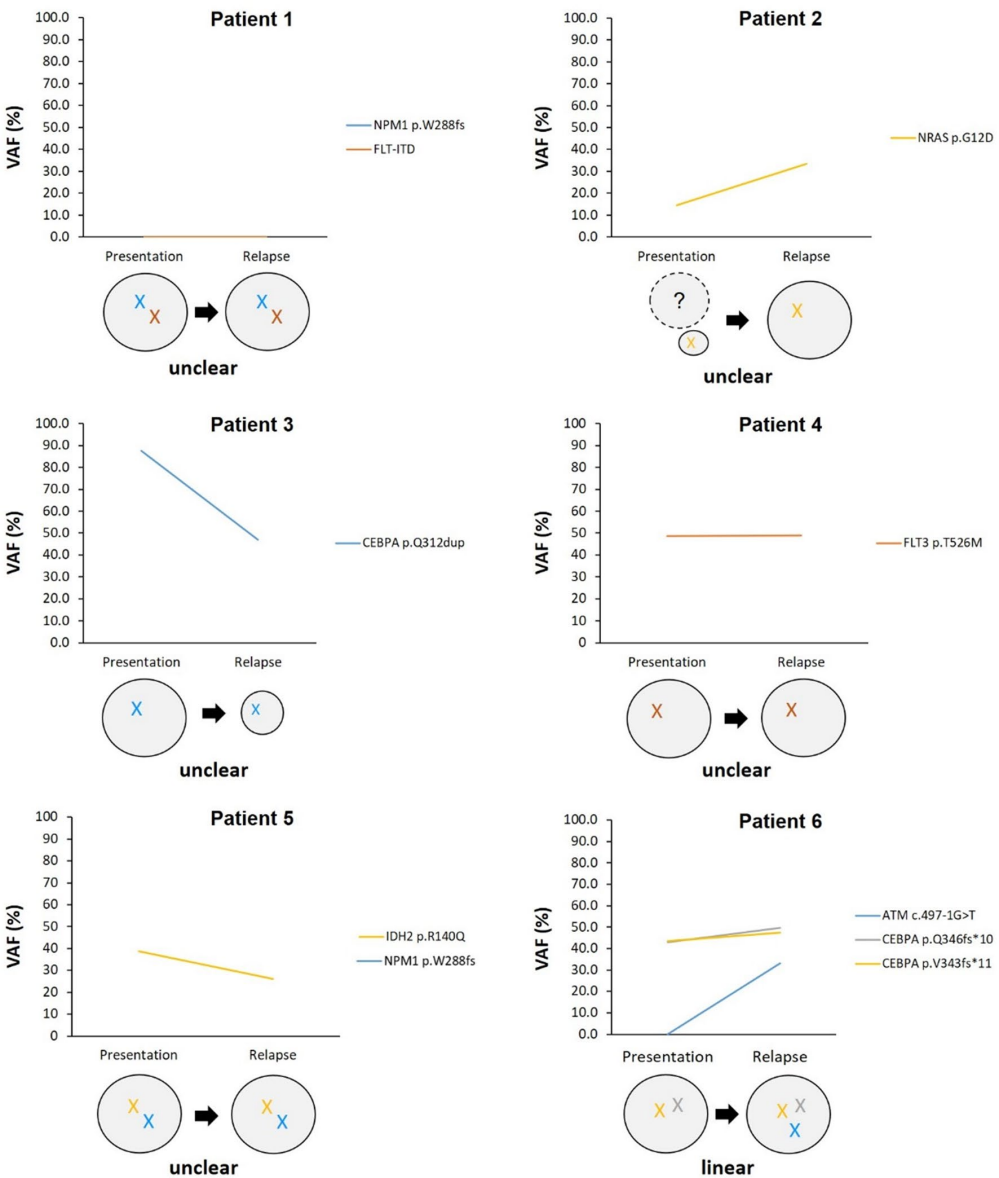
Clonal evolution in a single patient based on comparison between variant allele fraction (VAF) score at presentation and relapse. Each line corresponds to an individual mutation and illustrates the presence of the mutation at both time points. Each circle corresponds to an individual cell clone, defined by harboring the identical set of mutations

Variant allele fraction (VAF) was used to track each somatic variant from presentation to relapse to map clones that persisted, those that resolved from presentation to relapse as well as novel mutations that emerged at relapse in order to delineate the clonal architecture of leukemic cells and to identify driver mutations that promote clonal expansion in relapse AML (Fig. 4). *NRAS* p.G12D (Patient 2), *CEBPA* p.Q312dup (Patient 3), *FLT3* p.T526M (Patient 4), *IDH2* p.R140Q (Patient 5), *CEBPA* p.Q346fs*10 (Patient 6) and *CEBPA* p.V343fs*11 (Patient 6) were among mutations in known AML driver genes that were retained after chemotherapy with VAF of 14.4%, 87.6%, 48.5%, 38.7%, 42.9 and 43.5%, respectively, in the presentation samples and 33.4%, 47.0%, 48.8%, 26.1%, 49.6 and 47.4% in the relapse samples. On the other hand, ATM c.497-1G>T (Patient 6) was the only mutation in known AML driver genes acquired at relapse with VAF of 33.3%. VAF score for two mutations at both presentation and relapse in Patient 1 (*NPM1* p.W288fs and *FLT3*-ITD) and one mutation at presentation in Patient 5 (*NPM1* p.W288fs) detected by molecular diagnostic screening, were not available as they were not detectable by exome sequencing. In addition, mutations in other genes acquired at relapse include *PTPRQ* c.6452–6453 + 2del (Patient 1), *DNAH5* c.2578-2delA (Patient 2), *MAP3K20* c.416-2 A>T (Patient 4), *MAP3K20* c.416-1G>T (Patient 4), *WRN* c.840-2_841delAGGG (Patient 5), *WRN* p.S282fs*24 (Patient 5), *ATRX* p.R2407* (Patient 6) and *RYR2* p.T4580M (Patient 6) with a range of 15.2–80.0% VAF score (Table 1).

## Discussion

Cytogenetic findings have contributed to the understanding of genetic heterogeneity in AML, which plays an important role in establishing clonality and determining prognosis. However, this genetic heterogeneity is a major concern in cancer especially AML as it drives phenotypic adaptability which may contribute to clonal selection during treatment. One of the important causes of genetic heterogeneity is genomic instability. This instability leads to an increased mutation rate which can shape the evolution of the cancer genome through many mechanisms. Moreover, the diversity of genetic events in a cancer genome poses a significant challenge to identifying the oncogenic effect of specific genetic abnormalities. The advent of next generation sequencing (NGS) technology has allowed for more comprehensive analysis of somatic variants which has facilitated understanding of AML initiation and progression [10, 15–17, 20–22].

The present study compared the mutational landscape of 6 matched presentation and relapse CN-AML samples using high-throughput paired-end exome sequencing. The advantage of paired-end sequencing is that it allows for sequencing of both ends of a DNA fragment, resulting in high-quality and alignable sequence data that enables confident detection of genomic variants. However, one caveat with this approach is that genome coverage was very low (the human exome represents ~ 1.2% of the total genome) and mutations in non-coding sequence were not identified. Despite this, because of the high read depth achieved in this study as a result of exonic sequence enrichment, it was possible to identify somatic variants present in minor leukemic cell clones at presentation and relapse.

### Somatic variants at presentation and relapse

SNVs and indels are the most abundant type of genetic variation in the human genome with ratios of indels to SNVs were 0.19–0.22 in WGS and 0.14 in WES [23, 24]. In this study, the ratio of indels to SNVs was 0.60 at both presentation and relapse, with SNVs being approximately twice as common as indels. In addition, the average number of somatic variants (SNVs and indels) identified in this study were fewer compared to average number of mutations found in previous somatic mutation studies [16, 17, 21, 22, 25]. However, some previous studies had greater coverage of the genome (whole genome sequencing) and larger sample sizes whereas SNVs and indels identified in this study were strictly limited to somatic variants with potential biological implications in AML pathogenesis. Despite this, the number of somatic mutations in AML is relatively low with an average of 13 mutations per genome as opposed to other cancers occurring in adults which often have hundreds of somatic mutations, especially solid tumors such as breast, lung or pancreas [21]. Nevertheless, accurate identification of pathogenic SNVs and indels is one of the most important challenges in cancer genome analysis and requires detailed information of their impact on disease development. The use of analysis software equipped with linked databases that provides comprehensive information of each variant is very useful to discern functionality and a role in leukemogenesis.

### Coexisting variant within individual patients

The two-hit model of leukemogenesis has served as a basis to understand how mutations in certain genes drive AML [18]. Based on this model, mutations were classified according to two fundamental characteristics of cancer cells: mutations that confer cellular proliferation (class I mutations) and mutations that impair myeloid differentiation (class II mutations). The model predicts that both classes of mutation are required for AML transformation. However, genomic studies have revealed the presence of mutations in genes associated with epigenetic modification (*ASXL1*, *DNMT3A*, *IDH1/2*, *TET2*, *EZH2* and *MLL*) in a significant proportion of AML patients [26]. Disruption of epigenetic processes including DNA methylation and histone modification can lead to altered function in genes involve in key cellular pathways such as DNA repair, RAS signaling, cell cycle and apoptosis which may cause malignant cellular transformation in cancer [27]. In addition, a genome-wide DNA methylation profiling study has demonstrated that specific methylation profiles are associated with specific AML subtypes [28], which identified oncogenic cooperativity between somatic alterations in epigenetic regulators and known AML driver mutations. However, determining oncogenic cooperativity between mutations is difficult as it requires prior knowledge of the functions of the genes and the impact of the mutated genes on the translated protein. Therefore, given the abundant number of mutated genes with uncertain significance identified in this cohort, analysis was limited to the genes most recurrently mutated in AML as reported in COSMIC and to genes not previously implicated in AML pathogenesis but with significant functions potentially relevant for leukemogenesis.

Each patient in this study had a completely unique mutational spectrum, demonstrating the heterogeneous nature and complexity of the AML genomic landscape. Despite this variability, all patients had mutations in at least one known AML driver gene that is recurrently mutated in AML. *NPM1*, *FLT3*, *NRAS*, *CEBPA* and *IDH2* were the mutated genes identified in these patients at presentation which are well reported for their importance and contribution to AML pathogenesis. However, only one patient (Patient 1) presented with co-occurrence of class I and II mutations. The *FLT3*-ITD and *NPM1* p.W288fs (TCTG insertion) mutations are inarguably defined as AML driver mutations, as numerous studies have demonstrated their co-occurrence in AML patients [17, 20, 29, 30]. In Patient 3, *CEBPA* mutation appeared to be a driver mutation as it is recurrently mutated in AML patients [31, 32] and has been shown to play a key role in AML initiation in murine models [33, 34]. Although no additional mutations in known AML driver genes were detected in this patient, mutation in *MUTYH* gene appeared to cooperate with the *CEBPA* mutation at presentation due to its involvement in oxidative DNA damage repair [35, 36], compared to another accompanying mutation in the *CFTR* gene that was commonly reported in patients with cystic fibrosis [37, 38]. The *WRN* gene was recognized as a tumor-suppressor gene and evidence suggests that it plays a role in promoting oncogenic proliferation [39, 40] which may likely contribute to AML transformation in Patient 5, in addition to the *NPM1* and *IDH2* mutations.

Collectively, these findings emphasize the necessity of further investigation of mutation cooperativity, especially for mutations in genes that does not belong to either class I or class II mutations. Indeed, it is particularly important to identify mutation cooperativity in patients that lack known recurrent combinations of mutations as this might identify novel mechanisms of AML leukemogenesis.

### Clonal evolution from presentation to relapse

Indeed, flow cytometry is now routine practice in clinical laboratories for the determination of AML clonality essential to the diagnosis, prognosis and monitoring of disease as well as to direct treatment programs. However, one major advantage of NGS approaches over flow cytometry is the ability to quantify the proportion of variant reads for any given mutation, also known as the variant allele fraction (VAF), which indicates the percentage of tumor cells that harbor a specific mutation assuming a relatively pure leukemic sample. Using VAF score, each mutation at presentation can be tracked to map clones that persisted, those that resolved from presentation to relapse as well as novel mutations that emerged at relapse in order to delineate clonal evolution from presentation to relapse. A study demonstrated that multicolor flow cytometry (MFC) and NGS are complementary in detecting abnormal blast populations (via MFC) and somatic mutations (via NGS); however, a multigene NGS approach provides additional information on the mutational profiles of leukemic cells and aids in the better understanding of AML clonal evolution [41].

In most cases, mutations detected at presentation were also present at relapse with additional mutations, except for one case (Patient 3) which had no additional mutation acquired at relapse (Fig. 1). However, almost all of these additional mutations acquired at relapse are mutations in genes that have yet unknown function in leukemogenesis. By excluding these mutations, variants were analyzed to define the effect of chemotherapy on the prevalence of somatic variants at relapse specifically in known AML driver genes based on comparison between VAF score at presentation and relapse (Fig. 4). Except for *NPM1* p.W288fs and *FLT*3-ITD mutations in which no VAF scores were available (not detected by exome sequencing), *FLT3* p.T526M, *CEBPA* p.Q346fs*10 and *CEBPA* p.V343fs*11 mutations were found to be persist after treatment by having similar values at presentation and relapse (less than 10% difference). *NRA*S p.G12D mutation is predicted to confer relative resistance to standard combination chemotherapy treatment based on their increased VAF (greater than 10% difference) at relapse, whereas *CEBPA* p.Q312dup mutation was predicted to confer sensitivity to chemotherapy based on a reduced VAF score (greater than 10% difference) at relapse. On the other hand, *ATM* c.497-1G>T is predicted to be induced by or selected by chemotherapy since this mutation was not detected at presentation.

Further analysis was performed to determine the clonal evolution pattern from presentation to relapse in this cohort. Based on the models of clonal evolution of AML [42], a linear evolution pattern was clearly observed in one patient (epitomized by Patient 6; Fig. 4) which is characterized by the persistent of major clone at relapse with additional mutations. However, in some cases, relapse might be driven by the same set of mutations acquired at presentation, suggesting these mutations confer selective advantage during AML treatment, resulting in clonal expansion and eventually leading to relapse (epitomized by patient 1, 4 and 5; Fig. 4). In addition, despite the absence of additional mutations at relapse, the VAF score for *CEBPA* p.Q312dup was significantly decreased, suggesting a major clone with a *CEBPA* p.Q312dup mutation failed to survived chemotherapy (epitomized by patient 3; Fig. 4).

In summary, the findings from this study suggests two possible mechanisms of relapse: (1) a major clone at disease presentation survived chemotherapy and re-emerged at relapse after acquired additional mutations; (2) a major clone survived chemotherapy and re-emerged at relapse with the same set of mutations without additional mutations. The former showed that relapse is driven by the acquisition of additional somatic mutations, consistent with other previous studies investigating clonal evolution of AML [10, 15–17, 43], while the latter is associated with clonal evolution with stable mutations only [42], suggesting the presence of non-genetic alterations, such as epigenetic alterations, that might confer chemotherapy resistance. Sequence analysis of a remission sample is therefore essential to determine the VAF of these mutations at remission in order to confidently identify the pattern of clonal evolution. Furthermore, this study revealed significant variability in the mutational profile which demonstrates the heterogeneity of genomic landscape in AML; however, consists of classic AML driver and recurrent hotspot mutations as well as mutations in leukemia relevant genes. The mutational profile has important implications in treatment assignment, with a more precise, genomic guided therapy can be administered, thus limiting the use of genotoxic pro-mutagenic chemotherapy.

## Conclusions

The data from this study provide detailed information of mutations in CN-AML patients which is fundamental in identifying somatic variants with putative functional impact on AML pathogenesis, both at disease presentation and relapse, and facilitates better understanding of clonal evolution from presentation to relapse. Ultimately, this approach will allow for the identification of relapse-driver mutations that could be exploited for the development of synthetic lethal interactions and novel therapies to improve outcomes and reduce death from disease.

## Materials and methods

### Patient samples

A cohort of 6 matched presentation and relapsed CN-AML cases were retrospectively selected from the diagnostic cases within the National Referral Centre for Bone Marrow Cytogenetics and Molecular Genetics, Cancer Research Centre, Institute for Medical Research (IMR, Kuala Lumpur, Malaysia). Patients were selected based on the normal cytogenetic finding and the availability of DNA from the bone marrow samples with leukemic blasts > 20% at both disease presentation and relapse. Patients achieved clinical remission after treatment with combination chemotherapy that included ara-C and an anthracycline (daunorubicin or idarubicin), followed by consolidation chemotherapy that included high-dose of ara-C. The median age was 36.5 years (range: 17–51 years).

G-banding karyotype analysis of 20 metaphase chromosomal spreads from the bone marrow samples was performed to rule out numerical and structural chromosomal abnormalities. Multiplex RT-PCR using HemaVision®-28 N kit (DNA Diagnostic, Risskov, Denmark) was also performed for all samples to screen for 28 leukemia causing translocations including common translocations in AML such as t(8;21)(q22;q22), t(15;17)(q24;q21) and inv(16)(p13;q22) in order to exclude cryptic chromosomal abnormalities. This qualitative test uses reverse transcription of RNA to cDNA followed by multiplex nested polymerase chain reaction and agarose gel electrophoresis to identify chromosomes, genes and exons at the breakpoint in fusion genes. In addition, all samples were further screened for AML prognostic markers including gene mutations in *NPM1* and *FLT3* via targeted PCR using specific primers targeted to regions of interest. The characteristics and detailed information of these patients with cytogenetics and molecular profiles are further described in Table 3.

**Table 3:** Clinical and molecular characteristics of study cohort

Patient	Status	Age at Dx (years)	Blast%	G-banding Karyotype	Molecular diagnostic (28 translocations)	AML prognostic marker
1	P	26	55	46,XX [20]	Negative	*NPM1* (MutA) positive, *FLT3*-ITD positive
	R		30	46,XX [20]	Negative	*NPM1* (MutA) positive, *FLT3*-ITD negative
2	P	17	> 80	46,XX [20]	Negative	*NPM1* negative, *FLT3*-ITD negative
	R		60	46,XX [20]	Negative	*NPM1* negative, *FLT3*-ITD negative
3	P	43	91	46,XY [20]	Negative	*NPM1* negative, *FLT3*-ITD negative
	R		94	46,XY [20]	Negative	*NPM1* negative, *FLT3*-ITD negative
4	P	40	11	46,XY [20]	Negative	*NPM1* negative, *FLT3*-ITD negative
	R		70	46,XY [20]	Negative	*NPM1* negative, *FLT3*-ITD negative
5	P	51	54	46,XY [20]	Negative	*NPM1* (MutA) positive, *FLT3*-ITD negative
	R		20	46,XY [20]	Negative	*NPM1* (MutA) positive, *FLT3*-ITD negative
6	P	33	61	46,XX [20]	Negative	*NPM1* negative, *FLT3*-ITD negative
	R		91	46,XX [20]	Negative	*NPM1* negative, *FLT3*-ITD negative

### Whole exome sequencing

DNA from all primary patient samples was extracted using QIAamp DNA Blood Mini Kit (Qiagen) according to manufacturer’s protocol. Quantitation of the extracted DNA was carried out using a Nanodrop ND-1000 spectrophotometer and the required concentration and volume of DNA for exome sequencing was prepared according to sequencing service provider instruction. Sample preparation, library construction, sequencing and bioinformatics analysis were performed offsite by the Beijing Genome Institute (BGI) (Hong Kong, China). SureSelect Human All Exon V5 + UTR kit (Agilent Technologies, Santa Clara, USA) was used for enrichment of exon sequence including untranslated regions (UTRs). Samples were sequenced to give an average read depth (reads per base) of 100X, supporting the identification of minor leukemic cell clones. Paired-end sequencing was performed using Illumina HiSeq 4000 platform and analysis of raw data was processed according to BGI analysis pipeline to map reads against the hg19 build of the human genome and identify single nucleotide variants (SNVs) and insertion/deletions (indels). Paired-end whole exome sequencing was performed for each sample to identify somatic variants. By enriching for exonic sequence, high quality whole exome sequencing reads with average coverage of 93.8% of exome (range: 77.3–96.5%) and average sequencing depth/read depth of 151X (range: 15–434X) was achieved.

### Data analysis

Variant calling files (.VCF) were received from the BGI. Analysis of genetic variants was performed using Ingenuity Variant Analysis™ software (Qiagen Bioinformatics) to compare the somatic mutation profile of matched AML samples at presentation and relapse in order to identify somatic mutations that drive disease progression. A series of filters were applied to select variants most likely to impact on gene function or biological processes known to be implicated in disease progression. Variants with a read depth < 10 were excluded from the analysis as were variants with an allele frequency (VAF) ≥ 5% in the healthy population [databases used include the 1000 Genomes Project, ExAC, gnomAD, NHLBI ESP exomes; minor allele frequency (MAF) < 1%)]. Identification of putative AML driver mutations were determined by identification of variants in established AML genes. Specifically, the Catalogue of Somatic Mutations in Cancer (COSMIC) database was interrogated to identify the most frequently recurrently mutated genes in AML.

## Data Availability

Data available for sharing upon request.
